# Review of Optical Fiber Sensors for Temperature, Salinity, and Pressure Sensing and Measurement in Seawater

**DOI:** 10.3390/s22145363

**Published:** 2022-07-18

**Authors:** Honglin Liang, Jing Wang, Lihui Zhang, Jichao Liu, Shanshan Wang

**Affiliations:** Optics and Optoelectronics Laboratory, School of Physics and Optoelectronic Engineering, Ocean University of China, Qingdao 266100, China; lianghonglin@stu.ouc.edu.cn (H.L.); wjing@ouc.edu.cn (J.W.); zhanglihui@stu.ouc.edu.cn (L.Z.); liujichao@stu.ouc.edu.cn (J.L.)

**Keywords:** optical fiber sensor, temperature, salinity and pressure, ocean detection

## Abstract

Temperature, salinity, and pressure (TSP) are essential parameters for the ocean. Optical fiber sensors (OFSs) have rapidly come into focus as an ocean detection technology in recent years due to their advantages of electromagnetic interference, light weight, low cost, and no waterproof requirement. In this paper, the most recently developed TSP sensors for single parameter and multi-parameter TSP sensing and measurement based on different OFSs are reviewed. In addition, from the practical point of view, encapsulation methods that protect fibers and maintain the normal operation of OFSs in seawater, and the response time of the OFS, are addressed. Finally, we discuss the prospects and challenges of OFSs used in marine environments and provide some clues for future work.

## 1. Introduction

There is a long history of researching and exploring the ocean because it is an important supporting system for the human environment and contributes to the changes of the Earth’s environment and climate. The behavior of the ocean can be predicted through various parametric analyses. Generally, ocean parameters include physical, chemical, geographical, and biological parameters [1]. Precise values of parameters such as temperature, salinity, and pressure (TSP) will provide fundamental data for scientific researchers. For example, TSP data can not only be used as biogeochemistry and ecosystem background physical parameters [2,3,4], but also provide necessary compensation for calculating ocean acoustic transmission and other marine sensors.

For wide-range measurements of temperature and salinity (TS), data obtained from infrared or microwave satellites are available [5,6]. However, due to the limited penetration depth of infrared radiation and microwaves, they can only be used for obtaining the sea surface TS. What is more, the correction of remote sensing data also needs the support of in situ measured data [7,8]. The lack of real-time observation data has been restricting the development of marine scientific research.

To solve this problem, atmospheric and oceanographic scientists from the United States, Japan, and other countries proposed the global ocean environment observation project ARGO (Array for Real-Time Geostrophic Oceanography) project in 1998, which ushered in a new era in the field of marine environmental observation [9]. After nearly 20 years of joint efforts by countries around the world, a global ARGO ocean observation network composed of 3000 ARGO surface buoys has been built, which can continuously obtain the monitoring data of temperature, salinity, and depth to a depth of 2000 m in the deep ocean, and provide free data to scientists around the world for research and application. However, the buoy does not cover all of the ocean and cannot provide TSP data in any area.

In 1966, the idea of using a guiding light in glass fibers was proposed by Kao and Hockham [10]. These fibers greatly increased our ability to engineer optical fields with low loss, high compactness, flexible redirection, and much larger light−matter interaction length, which also opened a new field for optical sensing—optical fiber sensing. One of the most successful sensing technologies so far, optical sensing technology emerged a hundred years ago and has been well-established for 50 years; however, optical fiber sensing technology today is still developing and expanding to new areas of application continually. During these years, it has been widely used in industry and in environment, biology, chemistry, and health monitoring [11,12,13,14].

In the above areas, environmental monitoring is one of the most important applications. In particular, optical fiber sensors (OFSs) for ocean detection have undergone rapid development owing to their significant advances in recent years, such as in situ measurement, immunity to electromagnetic interference, multiplex or distribution with high spatial resolution, light weight, low cost, and low waterproof requirement [15,16]. Although not mainstream technology for ocean detection at present, OFSs show special advantages to some extent. For example, when comparing the temperature−depth profile measured by an autonomous underwater vehicle, the spatial resolution of the profile measured in the same sea area by an optical fiber grating (FG) temperature sensor is much higher [17]. In addition, the fixed-point measurement of TSP is realized by the conductivity temperature depth system (CTD) traditionally, and the temperature/salinity-depth profile is obtained by XCTD (expendable conductivity temperature depth) or XBT (expendable bathythermograph). When a low-cost application is required, such as expendable application, OFSs with a much lower price than that of electric devices may show more significant advantages from the point of economic cost.

There are a number of review articles on application of OFSs [17,18,19,20,21,22,23,24,25], and some of them are closely related to OFSs used in ocean detection [1,15,23,24,25]. However, few of them are specific to TSP sensing and measurement. Considering the great significance of TSP sensing and measurement in marine environmental monitoring, we focus on the application of OFSs for TSP sensing and measurement in seawater in this paper. The sensing principle and important parameters that describe the performance of OFSs for TSP sensing are introduced first. Then, according to different sensing and measurement objects, such as temperature/salinity/pressure single parameter sensing and multi-parameter sensing, different techniques applied are introduced individually, including FG, surface plasmon resonance (SPR), interferometric sensors, microfiber sensors (MSs), and hybrid structure sensors. Moreover, this survey article also addresses the practical application of OFSs in Section 5, including sensor encapsulation, response times, and a report on a sea trial of OFSs. Finally, prospects and challenges of OFSs used in marine observation are discussed. We hope that some points in this paper can be helpful or realized in future studies.

Because many methods and techniques are involved in this review, Abbreviations is shown first to give all full names and abbreviations used in this paper for better reading. We have only used full names when they appear for the first time.

## 2. Sensing Principle and Performance Parameters of OFSs

### 2.1. Sensing Principle

Whether OFSs can realize the sensing and measurement of TSP in seawater mainly depends on the relationship between the TSP and refractive index (RI) of seawater, which follows the empirical equation [26]:

n (*T*, *S*, *P*, *λ*) = 1.3247 + 3.3 × 10^3^ *λ*^−2^ − 3.2 × 10^7^ *λ*^−4^ − 2.5 × 10^−6^*T*^2^ + (5 − 2 × 10^−2^*T*)(4 × 10^−5^*S*) + (1.45 × 10^−5^*P*)(1.021 − 6 × 10^−4^*S*)(1 − 4.5 × 10^−3^*T*). (1)

where *λ* is the wavelength of probing light, and *T*, *S*, and *P* are temperature, salinity, and pressure of seawater, respectively. Besides the above equation, there are also other empirical equations that describe the dependence of RI on TSP [27]. Therefore, essentially speaking, TSP sensing and measurement by most OFSs is realized based on the TSP change-induced RI change. However, when different parameters are measured and different geometric structures are used, the sensing principle is not exactly the same. In this paper, realization of temperature/salinity/pressure single parameter sensing and multi-parameter sensing with different techniques are introduced according to different detection objects.

### 2.2. Performance Parameters for OFSs

To evaluate OFS performance, parameters such as sensitivity, detection limit, dynamic range, and response time are usually employed.

Sensitivity is one of the most important factors for OFSs used in ocean detection, and is defined as the ratio of the change in the measured optical parameter to the change of TSP to be measured [28]. Taking the interferometric devices used in temperature sensing, for example, there are two sensing sensitivity mechanisms generally: the resonant-wavelength-shift scheme and the intensity-variation scheme [29]. The former scheme monitors the shift of a resonant wavelength caused by TSP change. Correspondingly, temperature sensitivity is defined as $S_{T}=\frac{\partial \lambda }{\partial T}$. The latter scheme monitors the output intensity variation of a certain wavelength with TSP change and its temperature sensitivity is defined as $S_{T}=\frac{\partial I}{\partial T}$ where *λ* is the wavelength of probing light and *I* is the measured light intensity.

The detection limit is another important parameter to evaluate OFSs with definition of the minimum detectable signal change caused by the analyte [30], which is usually decided by sensitivity, system resolution, and some noise sources.

The dynamic range of OFSs used in TSP sensing and measurement usually refers to the range of TSP changes that can be detected by the sensor. Generally speaking, the TS ranges of seawater around the world are about −2~30 °C and 0~35‰ [1]. Further, considering a change of 1 MPa pressure corresponds to a depth change of about 0.1 km in the ocean, the dynamic range of pressure measurement on MPa level is required.

Response time (or time constant) is especially important for an OFS used for measurement of oceanic transient processes, such as the turbulence process [31]. In addition, when a sensor is used as a disposable sensor, the fast falling of the sensor in seawater also requires a fast response. Traditionally, temperature−depth profile measurement with XBT or XCTD is realized by a platinum resistor or a thermistor with a response time of about 65 ms (when the external flow velocity is 1 m/s) [32]. In other words, for OFSs used in marine applications, comparative or even faster responses should be achieved.

## 3. OFSs for Single-Parameter Sensing and Measurement in TSP

### 3.1. Temperature

Many types of OFS can be used for temperature sensing and measurement, such as FG, SPR, interferometric OFS, MS, and hybrid OFSs. We introduce temperature sensing and measurement with different devices used individually in this section.

#### 3.1.1. Temperature Sensing and Measurement Based on FG

FG is one of the most commonly used sensing devices, and is a periodic passive filtering device that makes the refractive index (RI) of the fiber core undergo axial periodic modulation to form a diffraction grating. According to the length of the written grating, FG can be divided into short-period fiber Bragg grating (FBG) and long-period fiber grating (LPG).

In recent years, FBG sensors have been widely used to measure various ocean parameters, and their structures and encapsulation methods are more and more diverse. Generally, bare FBG is sensitive to ambient temperature and pressure (TP), which can realize the sensing of seawater TP. In terms of temperature sensing and measurement by FBG, Wang et al. proposed a fast response ocean temperature sensor based on FBG in 2014 with a response time of 48.6 ms and a temperature sensitivity of 27.6 pm/°C [33]. In 2016, Ameen et al. developed a graphene diaphragm-integrated silica FBG sensor head for temperature measurement [34], which includes two FBG sensors made of SMF-28 fiber with central wavelength of 1550 nm, and a temperature sensitivity of 13.31 pm/°C.

Especially for LPG, Lan et al. proposed a high-order-mode LPG fabricated by point-by-point CO_2_ laser irradiation technology as early as 2011 [35]. In this way, it is easy to produce a turn-around-point (TAP) LPG that works in a variety of circumstances by adjusting the inscriptions period, by which a tenth order LPG was fabricated and demonstrated for temperature sensing. For example, in 2022, Berruti et al. demonstrated a well-designed TAP LPG and spliced it to a 150 nm thick Au-coated FBG, by which temperature sensitivity of 12.83 pm/°C was realized [36].

#### 3.1.2. Temperature Sensing and Measurement Based on SPR

SPR refers to the evanescent waves on fiber surfaces and the plasma exciter effect in metal films deposited on optical fibers. Generally, this effect is produced by coating a metal film on the surface of the fiber, which also requires wave vector matching conditions between the fiber evanescent wave and the surface plasmon wave [37].

Usually, SPR is used to measure multiple parameters simultaneously. Few of them are used for single parameter measurement. Zhao et al. realized a fiber-optic SPR-based temperature sensor with sensitivity of 1.5745 nm/°C in 2015 [38]. The sensing element was fabricated with a fiber probe coated with a silver layer, which was inserted into a capillary filled with thermosensitive anhydrous ethanol.

#### 3.1.3. Temperature Sensing and Measurement Based on an Interferometric OFS

The optical fiber interference measurement method is a conventional method that uses the interference principle of light for sensing. It refers to the interference of two or more beams. Sensing or measurement is realized by estimating the phase difference between the combined beams. Generally, interferometric sensors include the Fabry−Perot interferometer (FPI), the Mach−Zehnder interferometer (MZI), the Sagnac interferometer (SI), etc.

The FPI is often used for temperature sensing with the help of silicon wafers. Benefiting from the unique properties of silicon, such as large thermal diffusivity, notable thermo-optic effects and thermal expansion coefficient, this kind of sensor exhibits relatively high temperature sensitivity and very fast response [39]. For example, Hou et al. proposed a silicon wafer-based FPI in 2015, which achieved temperature sensitivities of 84–84.6 pm/°C and response time of about 2 ms, respectively [40,41].

**Figure 1:**
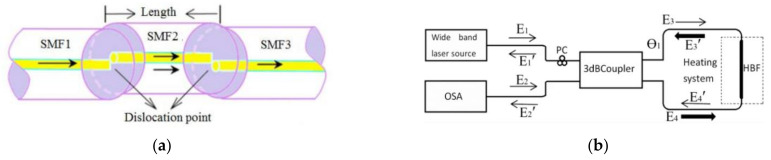

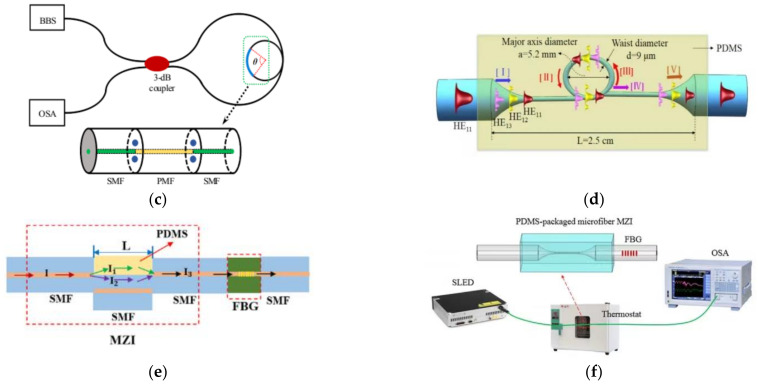
(**a**) Schematic diagram of the dislocation fiber MZI-based sensor [48]. (**b**) Schematic of HBEF sensor based on a Sagnac loop [51]. (**c**) Schematic diagram of an SI based on a PMF with a circle [53]. (**d**) Diagrammatic sketch of the PDMS-coated microfiber sensors [60]. (**e**) The sensing probe with cascaded MZI and FBG [62]. (**f**) Schematic of temperature measurement system [63].

MZI has been widely used in the sensing of a variety of physical parameters, including vibration [42], pressure [43,44], temperature [45], current [46], etc. For parameters in the ocean, MZI is generally used for TS sensing. In terms of temperature sensing, Zhao et al. designed a temperature sensor based on up-taper and single mode-multimode fiber (MMF) in 2016 [47]. The sensor splices SMF with a section of MMF by fusing an up-taper of the SMF. Within the temperature range of 20–80 °C, temperature sensitivity of 89.42 pm/°C can be achieved. The next year, Yan et al. fabricated a temperature MZI sensor based on an S-shaped dislocated optical fiber [48]. This sensor has two offset splicing points and makes the optical fiber between splicing points into an S-shaped micro bending (Figure 1a). The temperature sensitivity of the sensor is 50 pm/°C near the wavelength of 1545 nm. In contrast to the above two structures, Zhao et al. proposed a high-sensitivity temperature sensor with a wedge-shaped open cavity in 2021 [49]. The open-cavity realizes the direct contact between the sensing optical path and the external environment, and ensures the high sensitivity of the structure. The temperature sensitivity of the proposed sensor structure can reach 1.5 nm/°C. When it is filled with silicone oil, temperature sensitivity of 6 nm/°C can be obtained.

The SI is generally used to measure a single parameter. For example, Zhang et al. proposed an SI based on a short section of panda fiber for temperature measurement in 2011 [50]. With a commercial polarizer and polarization controller, the panda fiber provides a high sensitivity of −1.46 nm/°C and linear spectral response to the temperature change. Later, Wang et al. proposed an OFS based on a high birefringence elliptic fiber (HBEF) SI (Figure 1b) to measure single-point or continuous two-points seawater temperature with sensitivities of −472 pm/°C and −245 pm/°C, respectively [51,52]. In 2018, Shao et al. used an SI based on polarization maintaining fiber (PMF) to measure seawater temperature [53]. The SMF-PMF-SMF structure is bent into a circle with different radii in a Sagnac loop (Figure 1c). The maximum temperature sensing sensitivity can reach 1.73 nm/°C.

#### 3.1.4. Temperature Sensing and Measurement Based on MS

In recent years, OFSs fabricated by micro/nanofiber have attracted a lot of attention due to their great advantages of small size, nonlinear effect, strong surface intensity, and strong evanescent effect, and have been widely used in gas, liquid, force, bio-sensing, and other sensing fields [11,54,55,56,57]. Especially, the small size and strong evanescent field of microfibers offer an opportunity to develop TSP single/multi-parameter sensors with high sensitivity and fast response.

As early as 2014, Yang et al. demonstrated a seawater temperature sensor based on the microfiber knot/loop resonator (MKR) with sensitivities of 22.81 pm/°C [58]. On this basis, temperature measurement of seawater at two points can be achieved by cascading two MKRs [59]. In addition, Cai et al. designed a polydimethylsiloxane (PDMS) embedded MKR (Figure 1d) [60]. By Vernier effect, high temperature sensitivity of about −3.5 nm/°C is reached. In addition, Dang et al. designed an MKR encapsulated in PDMS with sensitivity of −8.48 nm/°C in 2020 [61]. By polymer encapsulation, not only is the sensitivity improved but the stability of the MKR is also improved significantly.

#### 3.1.5. Temperature Sensing and Measurement Based on a Hybrid OFS

For ocean exploration, a single sensor structure often fails to achieve ideal results, with problems such as low sensitivity, insufficient detection range, and so on. So, hybrid OFSs have been developed, by which not only the sensitivity may be improved, but also a larger measurement range and measurement accuracy may be achieved. For single parameter sensing, cascaded FBG and MZI sensors can be used to realize temperature sensing. For example, Tong et al. designed a high-sensitivity and wide measurement-range temperature MZI sensor based on three parts: a light lead-in fiber, a light lead-out fiber, and an offset fiber, and PDMS coated on the core-offset region (Figure 1e) [62]. Meanwhile, FBG was combined to enlarge the measurement range. The sensitivity of this sensor can reach 10.389 nm/°C from 10 to 59.4 °C. In the same year, they tuned the cascaded structure to work at the dispersion turning point and very high temperature sensitivity of 38 nm/°C was reached within the temperature range of 2.28 °C to 38.38 °C (Figure 1f) [63].

### 3.2. Salinity

Salinity is also one of the important physical parameters in the ocean. The commonly used fiber-optic salinity sensors also include FG, interferometric OFS, and MS types. In this section, salinity sensing and measurement based on these devices are described.

#### 3.2.1. Salinity Sensing and Measurement Based on FG

Usually, FBG without coating is only sensitive to TP. By coating specific materials on FBG, salinity sensing can be realized and the problem of cross sensitivity of multi-parameters can be solved. In addition, LPG can also be used to measure ocean salinity. In 2017, Yang et al. demonstrated a highly sensitive fiber-optic salinity sensor synergistically combining LPG and stimuli-responsive polyelectrolyte multilayers, with sensitivity of 36 nm/M [64]. Soon after, they demonstrated a highly sensitive and fast-response-time fiber-optic salinity sensor that incorporates LPG and ion-strength responsive hydrogels with sensitivity of 125.5 pm/‰ [65]. However, the long-term stability of the sensitizing coating remains to be further investigated.

#### 3.2.2. Salinity Sensing and Measurement Based on an Interferometric OFS

**Figure 2:**
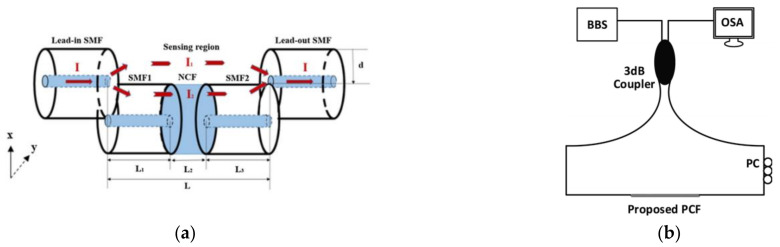
(**a**) Schematic diagram of an FPI based on offset splicing [68]; (**b**) SI generalized setup [70].

MZI and SI are interferometric fiber optic sensors commonly used in measuring salinity individually. In terms of MZI, Meng et al. proposed a sensor based on the multimode interference effect in 2013 [66]. The sensor was fabricated by splicing a section of no-core fiber (NCF) between two SMFs. Within a salinity range of 3.86–21.62%, the sensitivity was 19.4 pm/%. In 2019, Wang et al. proposed a kind of MZI based on an exposed-core micro-structured optical fiber, which used an exposed-core micro-structured optical fiber to realize salinity sensing [67]. The salinity sensitivity measured was about −2.29 nm/‰ within the salinity range of 0–40‰. In 2020, Lin et al. introduced a sensor based on the SMF-SMF-NCF-SMF-SMF structure (Figure 2a). In an environment with fixed temperature, its salinity sensitivity reached −3.42 nm/‰ within the range of 0–40.001‰ [68].

Wu et al. proposed a photonic crystal fiber (PCF) salinity sensor based on an SI in 2011 [69]. The SI was mainly composed of a 3dB coupler and a small section of Hi-Bi PCF coated with polyimide (PI), which realized salinity sensitivity up to 0.742 nm/(mol/L). Later, Md. Aslam Mollah et al. proposed a PCF salinity sensor based on an SI in 2020 (Figure 2b) [70]. Within the salinity range of 0–100%, the salinity sensitivity was 7.5 nm/%.

It is worth noting that PCF is a kind of special fiber (SF) that has periodically arranged microstructural holes in the cladding of the optical fiber. Its properties, such as potentially high birefringence, very small or high nonlinearity, low propagation losses, and controlled dispersion parameters, make it unique in many applications [71]. For example, Olyaee et al. introduced a nano-cavity photonic crystal resonator (NC-PCR)-based sensing platform for seawater salinity concentration detection in 2018 [72]. The results showed that the NC-PCR had high sensitivity and selectivity for different concentrations of seawater salinity.

#### 3.2.3. Salinity Sensing and Measurement Based on MS

Due to the relatively strong evanescent field effect and the direct contact measuring method, there is distinct advantage in salinity measurement and sensing using MS. MKR was first proposed and demonstrated in salinity sensing. As early as 2012, Wang et al. proposed a RI and salinity sensor based on MKR due to the thermo-optic and evanescent field effect of the microfiber [30]. Shortly after that, Liao et al. demonstrated seawater salinity sensors experimentally based on MKR with sensitivity of 21.18 pm/‰ [73]. To further improve the salinity sensitivity, Wang et al. proposed a salinity sensor with sensitivity as high as 2938.16 pm/‰ by non-adiabatic tapering a panda fiber to establish a multimode interferometer [74].

### 3.3. Pressure

Pressure measurement by OFSs is generally accompanied by temperature measurement, so there are few sensors that measure ocean pressure/depth separately. For example, Vaddadi et al. designed an FBG sensor that measured the pressure of ocean water in 2020 [75], in which a special design was introduced: stainless steel disks and rubber “O” rings were used in a closed air cavity setup. The pressure sensitivity could reach as high as 24.95 nm/MPa in a closed air cavity and 16.22 nm/MPa in an open-air cavity, respectively.

## 4. OFS for Multi-Parameter Sensing and Measurement in TSP

In comparison to single parameter measurement, multi-parameter measurement shows more practical value in ocean exploration. In this section, we introduce multi-parameter sensing and measurement, such as TS, TP, SP, and TSP sensing and measurement based on different techniques applied, respectively.

### 4.1. TS Dual-Parameter

Simultaneous measurement of TS by OFSs is the most common multi-parameter sensing and measurement in current studies, by which the fixed-point observation of TS in the ocean can be realized theoretically. Similarly, measurement of TS can also be realized by many different methods.

#### 4.1.1. TS Sensing and Measurement Based on FG

**Figure 3:**
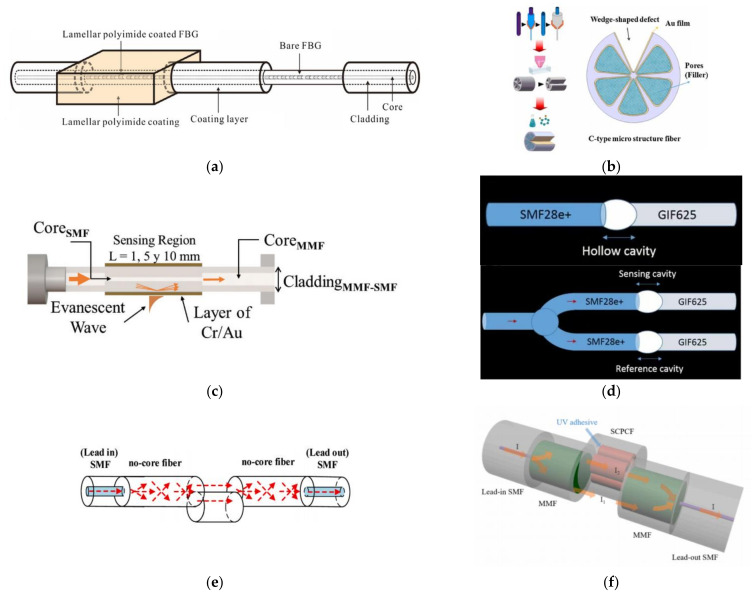
(**a**) Illustration of the multiplexed FBG sensing element [78]. (**b**) Production process of composite structure [79]. (**c**) Schematic diagram of SPR-based OFS [83]. (**d**) Schematic diagram of the FPI in single configuration and the Vernier sensor [91]. (**e**,**f**) Schematic diagrams of different FPIs based on offset splicing [93,94].

For two-parameter measurements, by cascading two polymer-coated FBGs, simultaneous measurement of TS was realized as early as 2008 [76]. In 2010, Men et al. used an FBG-based sensor system to measure salinity/sugar content and temperature [77]. The system consisted of two FBGs, one sensitive to salinity/sugar and the other to temperature. The sensitivity of salinity and temperature reached 0.0165 nm/M and 0.0094 nm/°C, respectively. Sun et al. proposed an FBG salinity sensor with temperature compensation for in situ monitoring of groundwater in 2019 [78]. The sensor consisted of an FBG coated with PI and an uncoated FBG (Figure 3a). The sensitivities of the coated FBG to salinity and temperature were 0.0358 nm/% and 0.0321 nm/°C, respectively.

#### 4.1.2. TS Sensing and Measurement Based on SPR

Usually, SPR fiber-optic sensors measure TS in seawater by means of combining fiber with different materials, such as metal or polymer. For example, Zhao et al. proposed a C-type micro-structured fiber for simultaneous measurement of TS in 2017 by moving a hole from a gold film-coated six-hole micro-structured fiber (Figure 3b) [79]. Experimental results showed that the maximum TS sensitivities were 1.402 nm/‰ and −7.609 nm/°C, respectively.

In addition to the indispensable metal film, SPR based OFS can also be combined with polymer material to further improve its performance, such as PDMS, which is usually used as a bio-layer in several OFSs [80,81,82]. For example, Velázquez-González et al. used gold-plated film-coated SMF (Figure 3c) to measure temperature and RI of a liquid sample in 2016 [83,84]. Sensitivities of 2664.540 nm/RIU and −2.852 nm/°C were realized in RI and temperature sensing. In 2018, Zhao et al. used cascaded Au-film coated SMF and MMF to realize the simultaneous measurement of seawater TS [85]. Sensitivities of TS sensing were −4.418 nm/°C and 0.0558 nm/%, respectively. In 2020, Li et al. proposed a reflective seawater salinity and temperature sensor with MMF-SMF structure [86]. This structure was also coated by Au film with sensitivities of 0.31 nm/‰ and −2.02 nm/°C. The following year, Wang et al. used PDMS encapsulated PCF to simultaneously measure salinity and temperature in seawater [87]. The salinity and temperature sensitivities of the sensor were 0.307 nm/‰ and −4.937 nm/°C, respectively. PCF is used for sensing because of its good sensing performance and unique stomatal structure, which can be filled with a variety of temperature-sensitive materials (not only PDMS) to achieve temperature sensing [88]. In addition to using special materials, some special properties of PCF, such as one-dimensional defects, can be used to measure both temperature and salinity [89].

#### 4.1.3. TS Sensing and Measurement Based on Interferometric OFSs

Interferometric OFSs can also measure multiple parameters simultaneously, such as FPIs, which use etching and splicing techniques, and MZIs based on SF. This kind of interferometric sensor, which can measure multiple parameters, sometimes needs special demodulation method to solve the sensitive cross problem between parameters.

FPI-based OFSs are usually formed by multiple reflectors in the form of fiber endfaces or other media. As early as 2011, Nguyen et al. realized simultaneous measurements of seawater TS using FPI structures [90]. The sensor consisted of etching a three-wave FPI in the SMF by focused ion beam (FIB). A few years later, Flores et al. demonstrated a TS sensor which was composed of bubbles manufactured by SMF (Figure 3d) and multimode graded index fiber [91]. The hollow cavity was fabricated by a combination of hydrofluoric acid (HF) etching and fusion splicing to act as F-P cavity. Through the analysis of peak shift in spectra, TS sensitivities of 13.91 ± 0.29 nm/M (or 1150.0 ± 24.0 nm/RIU) and −587.37 ± 0.01 pm/°C were achieved. In 2021, Zhao et al. proposed a structure using an offset splicing F-P cavity, which could also be used to measure the TS in seawater with sensitivities of 6.85 nm/°C and 50 nm/‰ [92]. By applying the frequency division multiplexing technical and cavity length demodulation technology, demodulation of the signal was realized in the full measurement range.

In addition, MZI can also measure TS in seawater simultaneously. Yan et al. proposed an open-cavity MZI based on NCF in 2020 [93]. This structure is fabricated by splicing a short section of NCF with a large offset between two sections of NCF (Figure 3e). The sensitivity is 3.444 nm/% in the range of 0.5–5% salt concentration. The temperature sensitivity of the sensor is 0.798 nm/°C. The next year, Zhao et al. proposed an optical fiber sensor based on a lateral offset mode interferometer using a suspended core PCF with temperature sensitivity of 1.486 nm/°C and salinity sensitivity of 2.495 nm/‰ (Figure 3f) [94]. The transfer matrix method is used to demodulate the signal.

#### 4.1.4. TS Sensing and Measurement Based on MS

Due to the evanescent field effect, microfiber sensors can be used as multi-parameter sensors naturally. In recent years, many microfiber sensors for simultaneous measurement of TS in seawater have been proposed and developed, such as OMC, MMZI, and MKR.

**Figure 4:**
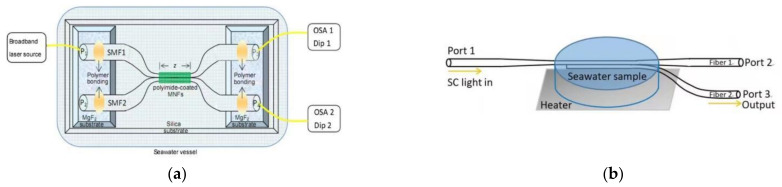

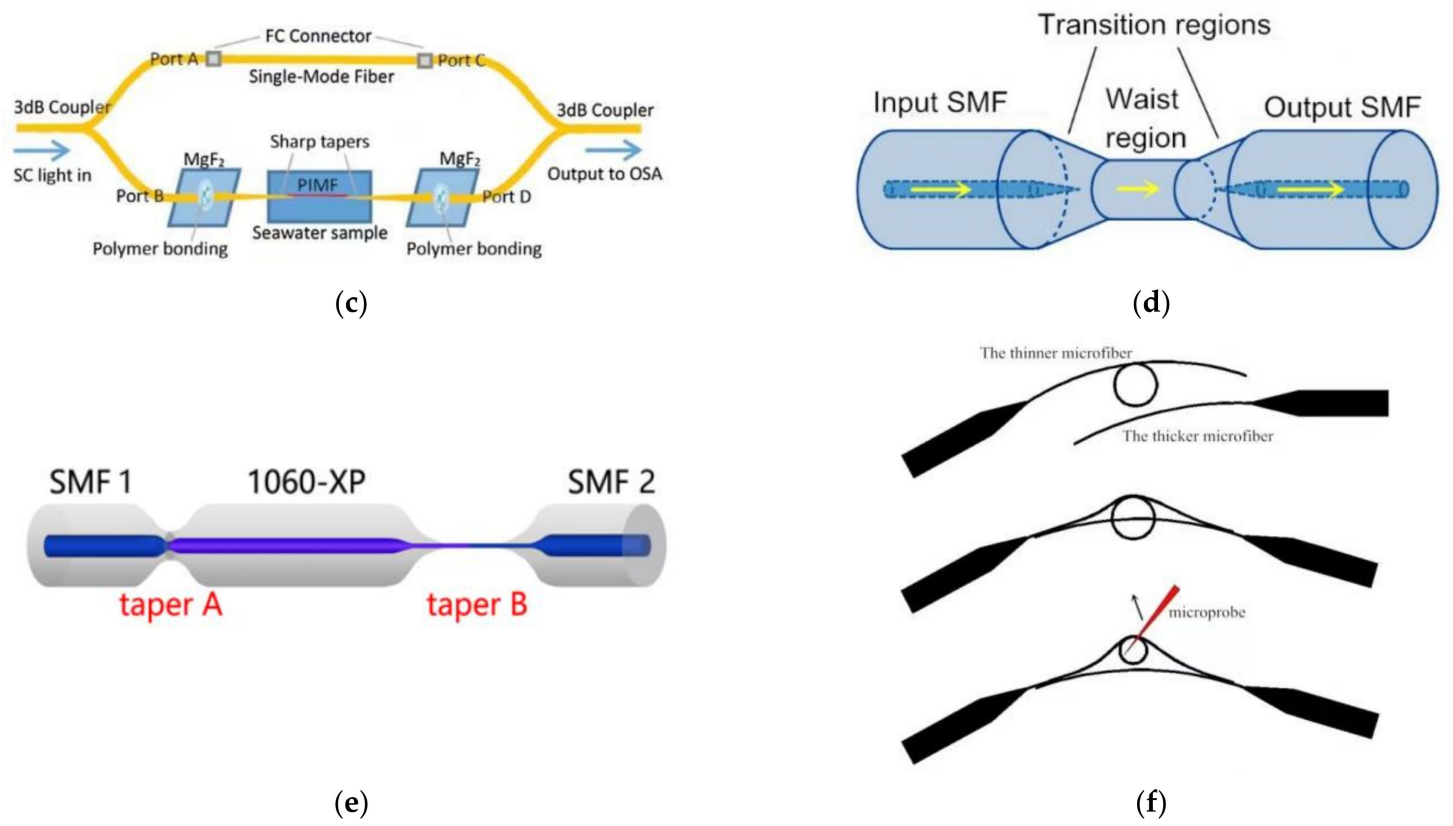
Schematic diagram of (**a**) the sensor [98] and (**b**) experiment setup [99] for the measurement of TS in seawater by OMC. (**c**) Schematic diagram of the hybrid MZI [102]. (**d**) Schematic diagrams of tapered fiber structure [103]. (**e**) Combining the in-line MMZI with tapered SF or Sagnac loop [104]. (**f**) The fabrication process of a microfiber MZ interferometer with a knot resonator structure [106]. Reprinted with permission from Ref. [106]. Copyright 2016 IEEE.

OMC consists of a fiber tapering (transition) region, a coupling region, and four input and output ports. The coupling region is composed of two parallel and tightly stacked/twisted microfibers. When the light is launched into the OMC, the two lowest-order even and odd modes are excited, and the energy will be periodically transmitted between the two modes in the coupling region. When the OMC structure is fixed, its transmission spectrum is completely determined by the RI of the surroundings. So, OMC was originally used for RI sensing [95,96,97]. In 2015, Wang et al. proposed a PI-coated OMC for TS sensing theoretically (Figure 4a) [98]. By monitoring the different sensing dips from two output ports simultaneously, measurement of seawater TS with high sensitivity was realized. Soon, TS measurements of seawater based on the OMC (Figure 4b) was demonstrated experimentally with maximum sensitivities of 930 pm/‰ and −1130 pm/°C, respectively [99]. By tracking two different dips, a two-by-two matrix is established and two parameters are demodulated. Based on the OMC, an OMC-Sagnac hybrid structure is developed by connecting two output ports together. By this structure, TS sensing is realized with sensitivities of 1007.4 pm/°C and 303.7 pm/‰ [100,101].

The MMZI is also a commonly used interferometric device, whose working principle is similar to those of an MZI assembled by SMF or SF. In 2018, a two-arm MMZI was proposed to realize the sensing of seawater temperature or salinity [102]; due to the PI material having a salinity−sensitivity feature and large thermal optic coefficient (TOC), a fluorinated PI microfiber is inserted into one of the arms (Figure 4c). The temperature and salinity sensitivities of this structure are 140 pm/°C and 64 pm/‰, respectively.

Compared with a two-arm MMZI, the in-line MMZI shows significant advantages of easy fabrication and encapsulation. Liao et al. proposed an ultra-high sensitivity MMZI sensor used for aqueous solution sensing (Figure 4d), which is obtained by the non-adiabatic tapering process [103]. Meanwhile, a universal sensitivity optimization equation is also derived. Taking the sensing of sodium nitrate in seawater, for example, an ultrahigh sensitivity of 14.95 pm/ppm is realized, whose equivalent sensitivity is about 1.26 × 10^5^ nm/RIU for RI sensing. In addition, by combining an in-line MMZI with tapered SF or Sagnac loop, simultaneous measurements of temperature and salinity are also achieved (Figure 4e) [104,105].

An MKR can also be combined with a microfiber Mach−Zehnder interferometer (MMZI) or sensitizing coating material to improve its multi-functionality or sensitivity. For example, Liao et al. realized the simultaneous measurement of TS in seawater by fabricating an MKR on one of the MMZI’s arms (Figure 4f) [106].

#### 4.1.5. TS Sensing and Measurement Based on Hybrid OFSs

Hybrid OFSs are generally used to measure multiple parameters. For example, cascaded MZI and FPI are generally used to measure TS simultaneously. In 2020, Zhao et al. proposed a sensor fabricated by asymmetric offset splicing [107]. At the offset interface, the light is divided into several parts, in which the transmitted light forms the MZI spectrum and the reflected light forms the FPI spectrum. The salinity sensitivity of the sensor can reach −2.4473 nm/‰ within the range of 20–40‰; the temperature sensitivity is higher than 2.17 nm/°C in the range of 28–48 °C. In the following year, they integrated an MZI and an FPI in the same channel to form a reflective fiber probe [108]. The sensing area is fabricated by offset splicing and the reflected light is greatly improved by special sputtering technology. Salinity sensitivity of the sensor can reach 2.7 nm/‰, and the temperature sensitivity is higher than 0.4 nm/°C. In 2022, this group proposed another sensor composed of an FPI and an MZI [109]. The femtosecond laser micromachining technology was used to modify the SMF and inscribe three consecutively connected waveguides. The modified area is etched by HF to form an inner channel of the fiber. Salinity and temperature sensitivities of the sensor are −2.323 nm/‰ and 2.176 nm/°C, respectively. In the same year, they used a section of hollow-core fiber with a U-shaped defect spliced between SMFs to form an FPI. The MZI is composed of laser-induced optical waveguides written in the cladding of the coating-retained SMF [110]. The maximum salinity and temperature sensitivities of the sensor are 0.244 nm/‰ and −2.767 nm/°C, respectively.

### 4.2. TP Dual-Parameter

It is easy to understand that when the TP in seawater can be measured simultaneously, a temperature−depth profile can be obtained theoretically. Therefore, OFS for TP measurement might be useful for developing some new optical methods to obtain the temperature−depth profile in seawater.

#### 4.2.1. TP Sensing and Measurement Based on FG

**Figure 5:**
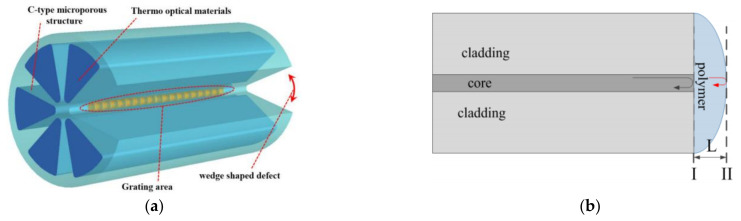

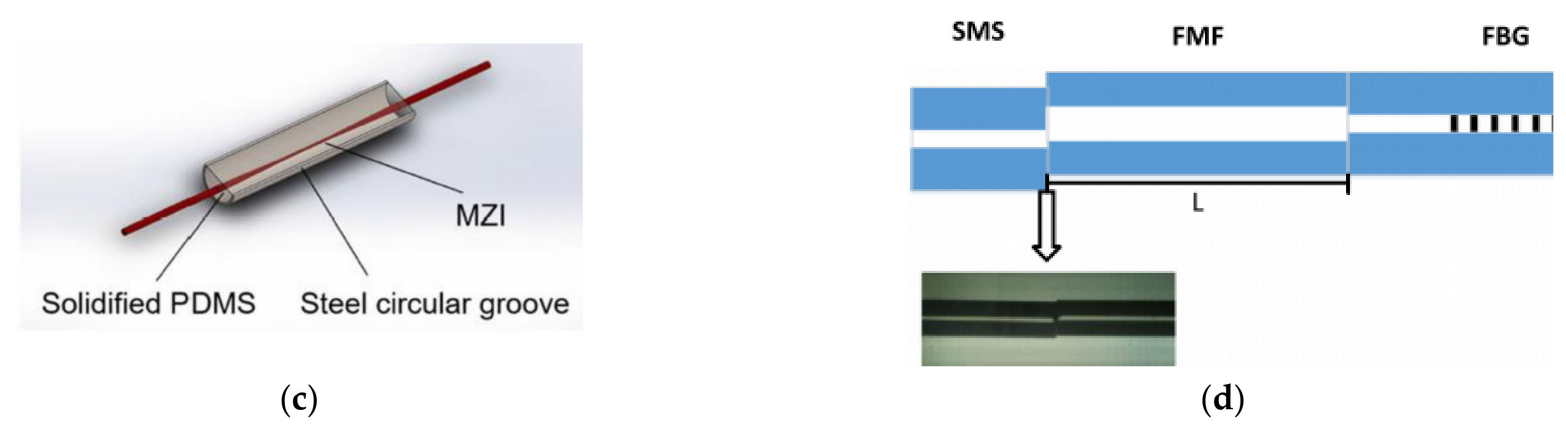
(**a**) Structure design of C-type micro-structured FG [111]. (**b**) Schematic diagram of the fiber-tip FPI sensor [114]. (**c**) Schematic diagram of the all-encapsulated and semi-encapsulated MMZI [116]. (**d**) Diagram of the cascaded MZI with SF [118].

In addition to measuring sea TS simultaneously with FBG sensors, Wu et al. designed a C-type micro-structured grating structure for simultaneous measurement of pressure and temperature in 2019 [111]. This structure uses the C-type structure to excite birefringence at the core of the grating (Figure 5a) and to improve the temperature sensitivity by filling the pores with thermal sensitive material. Pressure sensitivity of the sensor is −1.709 nm/MPa in the pressure range of 0–10 MPa, and temperature sensitivity is 1.054 nm/°C in the temperature range of 0–40 °C. Besides, Zhao et al. designed an ocean temperature−depth profile measuring instrument with FBG-LPG cascade structure in 2017, which laid a foundation for its application in the marine environment [112,113].

#### 4.2.2. TP Sensing and Measurement Based on an FPI

Among interferometric sensors, FPI sensors can be used to measure seawater TP. For example, in 2015, Sun et al. investigated an ultra-compact polymer-capped FPI based on SMF-end polymer to realize TP sensing simultaneously [114]. This structure is coated by polymer as a cap at the end of the SMF (Figure 5b), and the sensor reaches TP sensitivities of 249 pm/°C and 1130 pm/MPa, respectively.

#### 4.2.3. TP Sensing and Measurement Based on MS

As mentioned above, benefiting from the evanescent field effect, simultaneous measurement of seawater TP can also be realized by microfiber sensors. Just in this case, it is desirable to avoid salinity sensitivity and to enhance TP sensitivity. So, some polymers with high TOC and high elasticity are used to cover the microfiber for this purpose, such as PDMS. For example, a PDMS-immersed OMC−Sagnac hybrid structure is proposed to realize simultaneous measurement of TP with sensitivities of −2.133 nm/°C and 3.416 nm/MPa [115]. Similarly, by immersing the whole in-line MMZI into PDMS, sensing of TP in seawater with high sensitivity can also be realized with sensitivities of −7.41 nm/°C and 13.31 nm/MPa, respectively (Figure 5c) [116].

#### 4.2.4. TP Sensing and Measurement Based on Hybrid OFSs

Cascaded structures can be used to measure the TP of seawater simultaneously. In 2017, Dong et al. proposed a compact optical fiber sensor based on an FPI with an integrated FBG [117]. The sensitivities of the FPI sensor for measuring pressure and temperature are 501.4 nm/KPa and 306.2 nm/°C, respectively. The introduction of the FBG can effectively prevent the cross-sensitivity of the FPI. In addition, Gao et al. proposed a sensor based on few-mode fiber (FMF) and FBG in 2020 [118]. The sensor unit is composed of a segment of SMF and FMF dislocation splicing (Figure 5d). The temperature sensitivities of the sensor are −34.3 pm/°C and 10.7 pm/°C, and the pressure sensitivities are −2 pm/με and 0.67 pm/με, respectively.

### 4.3. SP Dual-Parameter

Similarly, when the SP in seawater can be measured simultaneously, a salinity−depth profile can be obtained theoretically. However, there are not many OFSs that measure SP simultaneously because there is also a temperature response for most OFSs. For example, by plating gold film on SMF, Yang et al. proposed a single-mode-multimode-single-mode-multimode-single-mode (SMSMS) structure, which realized the simultaneous sensing of SP with sensitivities of 0.36 nm/‰ and −1.42 nm/MPa, respectively [119]. To avoid temperature-induced errors, the experiment kept the temperature at 20 °C.

### 4.4. TSP Multi-Parameter

When OFSs are used to measure TSP simultaneously, in addition to the design of multi-parameter sensing, there are also problems such as cross-sensitivity among multi-parameters and the consequent problems of low sensitivity and the complex demodulation method. So, compared with single parameter or two-parameter sensing and measurement, TSP sensors are relatively few.

**Figure 6:**
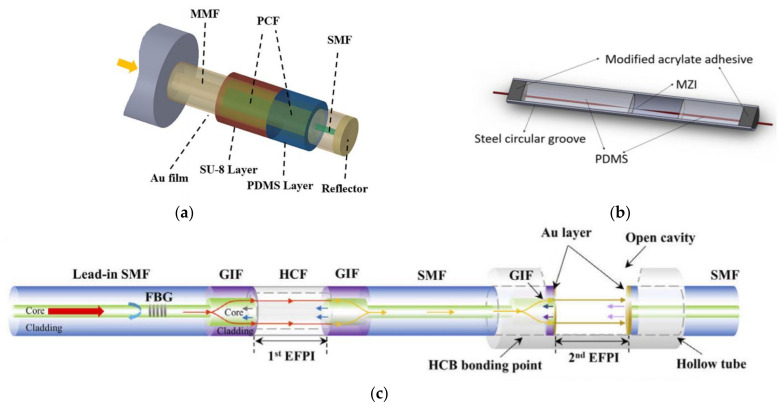
(**a**) Schematic diagram of an SPR-based OFS [121]. (**b**) Schematic diagram of the all-encapsulated and semi-encapsulated MMZI [123]. (**c**) Diagram of the cascaded MZI with SF [125].

Wang et al. developed an all-fiber CTD by cascading the FBG for pressure measurement and the LPG for TS measurement in 2012 with a precision of 0.01 °C for temperature measurement and better than 0.1% for pressure measurement. Salinity sensitivity is about 70.8 pm/‰ [120]. Subsequently, Zhao et al. realized the simultaneous measurement of TSP in seawater in 2019 [121]. In addition to the PDMS, they also used SU-8 to encapsulate the PCF with Au coating (Figure 6a). Experimental results show that the maximum sensitivities of TSP achieved to be 0.560 nm/‰, −1.802 nm/°C, and 2.838 nm/MPa, respectively. In the same year, Yu et al. demonstrated an all-fiber CTD based on an OMC sensor with TSP sensitivities of 2326 pm/°C, 1596 pm/‰, and 169 pm/MPa, respectively [122]. However, there were no comparisons with other commercial devices provided. In 2022, Liu et al. designed a TSP sensor based on the semi-encapsulated MMZI (Figure 6b) with TSP sensitivities of −2312 pm/°C, 631 pm/‰, and 3775 pm/MPa, respectively [123]. A new demodulation method based on machine learning for demodulating multi-parameters with cross sensitivity was also proposed and evaluated.

In terms of TSP sensing and measurement with a hybrid structure, Paladino et al. proposed a hybrid grating cavity to measure multiple parameters as early as 2010 [124]. This system consists of two FBGs: a uniform FBG and an FBG with a tilt angle. The sensor can measure the temperature, strain, surrounding RI, and bending characteristics of the structure with temperature and strain sensitivities of 9.5 pm/°C and 8.5 pm/g, respectively. In addition, Liu et al. made a fiber-optic probe for measuring TSP by using two extrinsic FPIs (EFPIs) and an FBG cascaded in an SMF in 2021 (Figure 6c) [125]. Using transfer matrix to minimize crosstalk, accurate demodulation can be achieved.

Finally, to make a comprehensive comparison of different TSP sensing and measurement methods, Table 1 was created to compare different sensors from the aspects of sensitivity, fabrication difficulty, and whether they have been tested in a real marine environment.

## 5. Practical Application of Optical Fiber Sensors in Ocean Observation

### 5.1. Encapsulation of Optical Fiber Sensors

Although the abovementioned OFSs have been developed adequately in the laboratory, it is necessary to develop an encapsulation method if we plan to use them for ocean detection. Encapsulation can not only increase the sensitivity, but also improve the robustness of the device. Generally, an OFS can be encapsulated by polymer, metal tube or capillary. For different sensing structures, due to their different sensing principles, their encapsulation methods are also not exactly the same.

**Figure 7:**
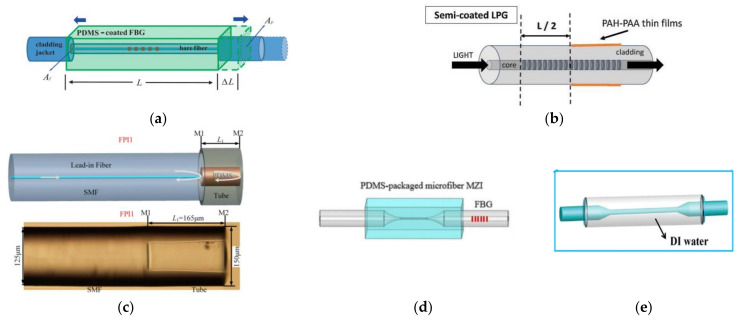
Schematic diagram of (**a**) PDMS-coated FBG [126]. (**b**) Semi-coated FBG [127]. (**c**) Encapsulation method of FPI sensors [131]. (**d**,**e**) Encapsulation method of MMZI sensors [62,133].

For FG encapsulation, there are many polymer encapsulation methods by embedding FG in polymer. For example, Park et al. encapsulated FG with PDMS (Figure 7a) to improve its sensitivity [126]. The results showed that the temperature sensitivity after encapsulation was 4.2 times higher than that of the exposed one. Urrutia utilized poly (acrylic acid) (PAA) and poly (allylamine hydrochloride) (PAH) to semi-encapsulate LPG (Figure 7b) to measure temperature and humidity with sensitivities of −410.66 pm/°C and −63.23 pm/%RH [127]. Metal film is used in FBG encapsulation as well as polymer. During the process of application, metal film is first deposited on the FBG and then welded with a metal tube.

In addition to the above single materials used, the hybrid encapsulation method using multiple materials has also been demonstrated. For example, FBG is supported on the elastic substrate and is covered with epoxy adhesive [128]. In addition, a diaphragm-type FBG pressure sensor and an FBG temperature sensor are developed to avoid the problem created by the disadvantages of the special FBG hybrid structure [129].

For interferometric sensor encapsulation, such as with an FPI, Li et al. inserted a SMF with flat end-face into a capillary to form an FPI [130]. Pan et al. proposed a temperature and pressure sensor with two cascaded cavities, one filled with PDMS and another coated with UV glue (Figure 7c) [131]. For most FPIs, their encapsulation relies on the cavity constructed by the end-face of the optical fiber and packaging tube.

For SPR sensor encapsulation, E et al. inserted the sensor head into a brass capillary and then filled it with PDMS [132]. Finally, the two ends of the capillary were sealed with UV glue, and temperature sensitivity of −1.87 nm/°C was achieved after encapsulation. In addition, Li et al. coated half of the surface of the SPR sensing unit with PDMS to realize the simultaneous measurement of seawater temperature and salinity with sensitivities of −2.02 nm/°C and 0.31 nm/‰ [86].

For microfiber sensors, due their thinner diameter, it is more necessary to encapsulate, in which polymers and capillaries are also used. Xia et al. encapsulated an MMZI sensor working at a dispersion turning point by PDMS (Figure 7d) with temperature sensitivity of about 38 nm/°C [62]. Wang et al. encapsulated part of the microfiber in the capillary and filled it with deionized water (Figure 7e) [133]. After encapsulation, the temperature sensitivity of the microfiber sensor is −415 pm/°C, which is six times higher than that of the un-encapsulated microfiber sensor. Hu et al. also encapsulated the part of the MMZI in a capillary and filled it with magnetic ionic liquid, and temperature sensitivity of −4.63 nm/°C was realized [134].

**Figure 8:**
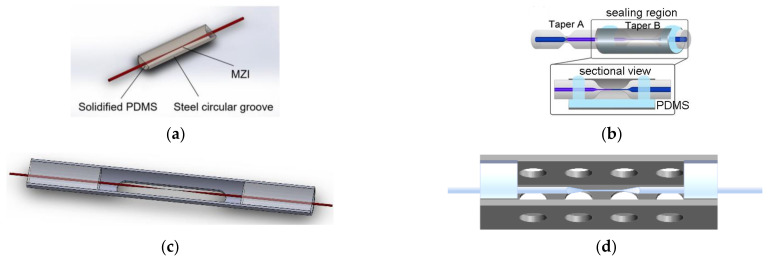
Schematic diagram of (**a**) full-encapsulated [116] and (**b**) part-encapsulated MMZI in a stainless steel C-type slot [104]. (**c**,**d**) Improved encapsulation method of MMZI [136].

In recent years, methods for encapsulating microfiber sensors used in an ocean environment have also been developed. For example, Hou et al. encapsulated MMZI in a stainless steel C-type slot with PDMS (Figure 8a) [116], in which PDMS completely covered the waist area and realized the simultaneous measurement of temperature and pressure. Liu et al. fixed both ends of MMZI with a U-shaped frame or added PDMS at the bottom and both ends of a C-shaped groove to encapsulate MMZI in the steel groove (Figure 8b) [31,104,135]. In this method, PDMS did not cover the waist and tapering areas and achieved the measurement of salinity.

Recently, Zhang et al. improved the above methods using 302AB glue or epoxy transparent AB glue to encapsulate the MMZI sensor in a side-opened steel groove (Figure 8c,d) [136], which greatly improved the nonlinear temperature response, the robustness, and response speed of the MMZI sensor. The results show that though the encapsulation protects the sensor, meanwhile, temperature sensitivity and response time may also be affected by encapsulation.

### 5.2. Response Time of Optical Fiber Sensors

As one of the most widely tested OFSs in sea trial, the response time of FBGs in temperature sensing has been investigated by researchers [33,137,138,139]. The results show that the response time of a bare FBG is about 20 ms in seawater [137]. When it is encapsulated, the response time will increase.

Compared with FBG sensors, microfiber sensors show a much faster response due to the larger surface to volume ratio. In 2020, Wen et al. measured the response time of a bare MMZI in temperature sensing with a result of 13.1 ms [31]. Similar to that of the FBG, when the microfiber is encapsulated, the response time will increase in varying degrees. For example, Liu et al. realized a response time of 33 ms in salinity sensing [104]; Hou et al. obtained response times of 1.13 s and 286 ms in TP sensing [116]. In the TP sensing, Lu et al. obtained response times of 2.6 s and 960 ms, respectively [115]. By optimizing the encapsulation, the response time after encapsulation of MMZI in TS sensing was about 219.0 ms and 12.5 ms, respectively [136].

### 5.3. Sea Trial of Optical Fiber Sensors

The ultimate goal of developing OFSs is to apply them in the marine environment for monitoring, and there have been more and more reports of point and distributed OFSs in recent years. Usually, a single-point sensor is used for monitoring the parameter at one point in the form of fixed-point observation and a distributed sensor is used for obtaining the temperature/salinity−depth profile perpendicular to the sea surface in the form of a sensor chain or expendable sensor.

In 2017, Dinesh Babu Duraibabu et al. presented an underwater single-point sensor based on a combination of an FPI and an FBG for measuring seawater pressure and temperature, respectively [140]. The pressure sensitivity and resolution of the sensor were 15 nm/kPa and 0.0784 kPa, respectively, and the temperature sensitivity and resolution were 12.5 pm/K and 0.1 °C, respectively. The sensor was entirely made of glass and sealed in a 316 stainless steel tube using Loctite Epoxy Marine. Sea trial results showed that there was a good agreement between the optical sensor and CTD (SBE 9Plus). In 2018, Chen et al. proposed a light RI sensor for seawater salinity monitoring. This is a compact RI sensor based on the total internal reflection method. In order to evaluate the applicability of the sensor in real turbid sea conditions, field performance tests of the infrared sensor were carried out during an ocean cruise in the east of the Yangtze River estuary in July 2017 [141]. The same year, an FBG sensor system was designed and attached on an underwater pressure vessel, then subjected to various diving depths up to 100 m, and the obtained maximum sensitivity was −22.364 με/bar [142].

When a single-point sensor is dropped and falls through the seawater, it measures temperatures at different depths. So, it can be used to measure a temperature−depth profile. For example, Zhao et al. proposed an all-fiber XBT (AOF-XBT) based on two cascaded FBGs to measure the seawater temperature−depth profile in 2022 [129]. The two FBGs were used as a temperature sensor and a pressure sensor, respectively. The average temperature sensitivity was 14.765 pm/°C and 13.705 pm/°C, respectively, in the temperature measurement range of 5–30 °C, and the average pressure sensitivity was −2.75586 nm/MPa and −3.00472 nm/MPa, respectively, in the pressure measurement range of 0–0.6 MPa. The sea test site was Weihai and the temperature accuracy was 0.01 °C. Compared with CTD (SBE 911PLUS) data, the measuring error was ±0.02 °C.

In addition, temperature−depth profile measurement can also be realized by a chain made up of cascaded FBGs or distributed sensor. For example, Li et al. proposed an FBG seawater temperature sensing system in 2011 [143]. The FBG sensor array system consisted of four 75 m optical fibers to carry 60 FBG temperature sensors. The sea trial was performed in the South China Sea. By comparing the measured results with CTD (YRS1-1), measurement accuracy better than 0.2 °C was obtained. In 2017, C. Garcia Izquierdo et al. also demonstrated a distributed temperature sensor monitoring system, in which an FBG was written into the single-mode fiber coated with acrylates (SM-ITU652) [144]. To enhance its environmental adaptability, the fiber-optic sensor was encapsulated in a 316L stainless steel tube with a seawater-resistant (polypropylene/PEEK) coating. The sea trial site was located in Vilanova I la Geltrú (Barcelona, Spain) with water depth of about 20 m. The measurement error was less than 0.1 °C compared with CTD. In 2020, Wang et al. proposed a high-spatial-resolution (5 m) sensor array for temperature and depth measurement based on an FBG [17]. The sensing chain consisted of 10 temperature sensors, a pressure sensor and a temperature compensation sensor. To eliminate the effect of temperature on the pressure sensor, EPO-TEK 353 ND glue was used to fix the temperature sensor in a metal tube so that it was not affected by external stress. This sensing chain was tested in the Yellow Sea with water depth of about 70 m. The temperature accuracy was 0.01 °C and the depth accuracy was 0.1% in full scale. Compared with the CTD (SBE56) data, the error was less than 0.05 °C.

Besides the above temperature chain composed of FBGs, Stefano Carlino et al. also proposed a temperature−depth profile monitoring system using stimulated Brillouin scattering in optical fibers in 2016 [145]. The cable was made up of six single-mode telecom- grade optical fibers submerged in the seabed, which was used to measure the temperature−depth profile near the active crater 2.4 km offshore of Campi Flighley (Volcano Bay). The maximum monitoring profile depth of the system optical cable was 85 m (the average depth was about 50 m) and the spatial resolution was 2 m. The measurement error was about 1 °C.

Compared with temperature and depth measurement, the sea trial reports on salinity sensors are relatively few. In 2006, Díaz-Herrera et al. proposed an SPR-based OFS for ocean salinity measurement [146]. The optical fiber sensor was encapsulated in an all-optical fiber probe and placed by the winch. The test was performed in three different modes in the Baltic Sea: a static test, a towing test, and a section test. The measured results were in good agreement with commercial CTD (IMCTD-MBP-D) data.

To make a comparison of different OFSs tested in sea trials, Table 2 was created to compare them from the aspects of sensing parameters and the obtained results.

## 6. Conclusions

In this paper, we have reviewed the development of several OFSs used for TSP sensing and measuring in seawater, and introduced the encapsulation, response time and sea trial application of typical fiber sensors. So far, a variety of OFSs for TSP sensing and measurement have been demonstrated. As mentioned above, some of them have already been tested in sea trials, such as FBG and FPI sensors. However, very few of them have migrated to the commercialized product level. “How to walk the last mile” becomes the key for the practical application of OFSs in the future. From this point, we can see that practical applications of OFSs for ocean detection still have a long way to go.

Especially for OFSs that have not been used in sea trial, it is urgent that we put them into practical use through reasonable encapsulation design because the purpose of developing sensors is to apply them in the actual environment. For an OFS that has been tested in sea trials, such as those for long-term fixed-point observations, adhesion and contamination problems have yet to be resolved, which is also a big problem for any sensor used in the ocean. In addition, the development of the array/distributed long-term measurement method has a great application for real-time monitoring of mesoscale ocean phenomena, such as the internal wave phenomenon, which can provide valuable data for inverting amplitude and propagation velocity of internal solitary waves, so as to afford data support for remote sensing.

In addition, for disposable optical fiber sensors, though the pollution problem can be temporarily ignored due to their short working time, there are also other problems to be solved. Because an OFS itself is very sensitive, discovering how to improve the repeatability and anti-interference is very important. In addition, for disposable FBG sensors, discovering how to expand their function and increase salinity sensitivity is very crucial. Finally, for microfiber sensors, how to improve the preparation repeatability of microfiber and the applicability of multi-parameter sensors in a changeable environment are also a great challenge.

As one of the most successful sensing technologies so far, besides the application of TSP sensing and measurement, optical fiber sensors will continue to open up opportunities in applications of flow rate sensors, ocean turbulence sensors, low concentration components (such as nitrate) sensors, acoustic sensors, and ocean internal wave measurement systems. We believe that the rapid development of new sensing structures, materials, and mechanisms for optical sensing will further progress the application of fiber sensors used in ocean detection, and the OFS will play more and more important roles in ocean detection in the future.

## Figures and Tables

**Table 1:** Comparisons of different OFSs for TSP sensing and measurement.

Type	Structure	S_T_ (nm/°C)	S_S_ (nm/‰)	S_P_ (nm/MPa)	Fabrication Difficulty	Sea Trial
FBG		0.0094 [76]–1.054 [111]	0.00358 [78]–0.0708 [120]	0.5 [120]–24.95 [75]	Medium	√
SPR		1.802 [121]–7.609 [79]	0.00558 [85]–1.402 [79]	1.42 [119]–2.838 [121]	Difficulty	√
Interferometric OFS	FPI	0.249 [114]–6.85 [92]	50 [92]	1.13 [114]	Medium	-
	MZI	0.05 [48]–6 [49]	0.0194 [66]–3.42 [68]	-	Easy	-
	SI	0.0149 [69]–1.73 [53]	0.75 [70]	-	Easy	-
Hybrid OFS	FBG MZI	0.0107 [118]–38.38 [63]	-	-	Medium	-
	FPI MZI	0.4 [108]–2.767 [110]	0.244 [110]–2.7 [108]	-	Medium	-
	FPI FBG	306.2 [117]	-	5.014 × 105 [117]	Medium	√
Microfiber	MKR	0.02281 [73]–8.48 [61]	0.02118 [58]	-	Medium	-
	OMC	1.0074 [100]–2.326 [122]	0.3037 [101]–1.596 [122]	0.169 [122]–3.416 [115]	Difficulty	-
	MMZI	0.14 [102]–7.41 [116]	0.064 [102]	13.31 [116]	Easy	√

√ Means that sea trials of these sensors have been reported.

**Table 2:** OFSs applied in sea trials.

Time	Location	Depth	Structure	Parameter	Result	Reference
2017	(49.54 N, 16.31 W)	14 m	FPI FBG	Pressure	Pressure resolution of 0.0784 kPa;	[140]
	(49.67 N, 16.37 W)			Temperature	Temperature resolution of 0.1 °C	
2021	Sea area of Weihai	70 m	two FBGs	Temperature−depth profile	Temperature error of ±0.02 °C	[129]
2018	-	100 m	FBG	pressure	–22.364 με/bar	[142]
2011	South China Sea	300 m	FBG array	Temperature−depth profile	Temperature accuracy of better than 0.2 °C	[143]
2017	Vilanova I la Geltrú coast	20 m	FBG	Temperature−depth profile	Temperature error of less than 0.1 °C	[144]
2020	Yellow Sea of China	70 m	FBG array	Temperature−depth profile	Temperature accuracy of 0.01 °C	[17]
					Depth accuracy of 0.1%	
2016	Volcano Bay	50 m	SMF	Temperature−depth profile	Temperature error of about 1 °C	[145]
2006	Baltic	-	SPR	Salinity	-	[146]

## Data Availability

Not applicable.

## Abbreviations

**Full** **Name**	**Abbreviation**	**Full** **Name**	**Abbreviation**
temperature, salinity, and pressure	TSP	temperature and salinity	TS
temperature and pressure	TP	salinity and pressure	SP
optical fiber sensor	OFS	microfiber sensor	MS
expendable conductivity temperature depth	XCTD	conductivity temperature depth system	CTD
expendable bathythermograph	XBT	refractive index	RI
special fiber	SF	single mode fiber	SMF
no-core fiber	NCF	multimode fiber	MMF
high birefringence elliptic fiber	HBEF	polarization maintaining fiber	PMF
photonic crystal fiber	PCF	few-mode fiber	FMF
thermal optic coefficient	TOC	turn-around-point	TAP
fiber grating	FG	surface plasmon resonance	SPR
long period fiber grating	LPG	fiber Bragg grating	FBG
extrinsic FPIs	EFPI	Fabry−Perot interferometer	FPI
microfiber Mach−Zehnder interferometer	MMZI	Mach−Zehnder interferometer	MZI
microfiber knot/loop resonator	MKR	Sagnac interferometer	SI
optical fiber coupler	OMC	nano-cavity photonic crystal resonator	NC-PCR
single-mode-multimode-single-mode-multimode-single-mode	SMSMS	polydimethylsiloxane	PDMS
poly (acrylic acid)	PAA	polyimide	PI
poly (allylamine hydrochloride)	PAH		
